# Supplementary material for: WordCommentsAnalyzer: A windows software tool for qualitative research
Source: F1000Res. 2018 Sep 4;7:536. Originally published 2018 May 3. [Version 2] doi: 10.12688/f1000research.14819.2 (PMC6137411; doi:10.12688/f1000research.14819.2)
Supplement: Supplementary file 3 [file f1000research-7-17614-s0002.tgz › b2a79496-4ada-47c4-9682-a99ce867836d.zip!/Eight Iranian Health Journals/Journal of Education and Community Health_0.docx]

© Journal of Education and Community Health. This work is licensed under CC BY-NC 4.0 (https://creativecommons.org/licenses/by-nc/4.0/).

Volume & Issue: دوره 5 - شماره 1

Title: سواد سلامت: مولفه کلیدی کنترل عوامل اجتماعی تعیین کننده سلامت

http://jech.umsha.ac.ir/article-1-486-fa.html

امروزه سواد سلامت به عنوان یک شاخص مهم و حیاتی در نتایج و هزینه های مراقبت های بهداشتی شناخته شده و لازمه نظام مراقبت های بهداشتی، داشتن سواد سلامت بالا می باشد . سواد سلامت به طور وسیعی به عنوان یک تعیین کننده سلامت و یک اولویت در دستور کار سیاست سلامت همگانی در نظر گرفته می شود. کم بودن سواد سلامت با سطح پایین سلامت، نابرابری های گسترده تر و هزینه های بالاتر نظام سلامت همراه است . سواد سلامت افراد را توانمند می سازد تا نقش فعالی در تغییر محیط ها برای تاثیرگذاری بر سلامت داشته باشند. این مفاهیم وسیع تر سواد سلامت، منعکس دهنده این دیدگاه است که سواد سلامت تنها یک مسئولیت فردی نیست. نظام های سلامت، خدمات و بخش های حرفه ای سلامت باید تسهیلات لازم برای ارتقای سواد سلامت را در سطح وسیع تر جمعیت فراهم آورند.

Title: ارزیابی اثر بخشی مدل مراقبت مشارکتی بر کیفیت زندگی و شاخص های متابولیکی بیماران مبتلا به دیابت نوع دو

http://jech.umsha.ac.ir/article-1-423-fa.html

سابقه و هدف: آموزش به بیمار مبتلا به دیابت نقش مهمی در بهبود کیفیت زندگی آنان دارد. مدل مراقبت مشارکتی از روش های نوین آموزش مراقبت از خود است، که در تحقیق حاضر تأثیر بکارگیری آن بر کیفیت زندگی و شاخص های متابولیکی بیماران مبتلا به دیابت نوع 2 مورد بررسی قرار گرفت. مواد و روش ها: این پژوهش نیمه تجربی از نوع پیش آزمون-پس آزمون است. 60 بیمار مبتلا به دیابت نوع 2 مراجعه کننده به درمانگاه دیابت بیمارستان شهر جیرفت در سال 1394-95 با نمونه گیری در دسترس انتخاب شدند. برای اجرای مدل مشارکتی که شامل چهار مرحله انگیزش، آماده سازی، درگیرسازی (اجرا) و ارزشیابی بود، بیماران به 4 گروه تقسیم و طی مدت 3 ماه برای هر گروه مدل مشارکتی اجرا شد. ابزار جمع آوری داده ها پرسشنامه بود. داده ها با استفاده از نرم افزار SPSS نسخه 20 و آزمون های آماری ویلکاکسون، مک نمار و  tزوجی مورد تجزیه و تحلیل قرار گرفتند. یافته‌ها: میانگین کلی کیفیت زندگی بعد از اجرای مدل مراقبت مشارکتی از 5.7±50.7 به 5.2±68.3 افزایش معنی داری نشان داد. نتایج آزمون ویلکاکسون تفاوت معنی داری را در شاخص های متابولیکی و مراقبتی نشان می دهد (0.001>P). همچنین ابعاد مختلف کیفیت زندگی افزایش معنی داری نسبت به قبل از اجرای مدل مراقبت مشارکتی نشان داد (0.001>P). نتیجه گیری: اجرای مدل مشارکتی باعث بهبود شاخص های متابولیکی و مراقبتی تاثیرگذار بر کیفیت زندگی می شود. لذا بکارگیری مدل مراقبت مشارکتی به عنوان یک مدل کارآمد و موثر در آموزش بیماران مبتلا به دیابت نوع 2 برای بهبود کیفیت زندگی توصیه می گردد.

Title: رابطه بین اعتیاد به اینترنت و افسردگی در بین دانش آموزان دوره متوسطه دوم شهرستان کردکوی با تأکید بر نوع شبکه های مجازی

http://jech.umsha.ac.ir/article-1-402-fa.html

سابقه و هدف: اینترنت می­ تواند به­ عنوان یک دستاورد مفید و اثربخش باشد و گاهی به دلیل استفاده نامناسب، بهداشت روانی کاربران را با تهدید روبرو می­ سازد و این امر به ­گونه ­ای است که در دهه ­های اخیر به­ عنوان یکی از عوامل اعتیاد مطرح شده است. یکی از اثرات این اعتیاد، ایجاد افسردگی بین افراد است. لذا، هدف پژوهش حاضر، بررسی رابطه بین اعتیاد به اینترنت و افسردگی در میان دانش آموزان مقطع متوسطه دوم می باشد. مواد و روش­ ها: روش پژوهش، توصیفی- پیمایشی از نوع همبستگی بوده است. جامعه­ آماری شامل دانش ­آموزان مقطع متوسطه دوم شهرستان کردکوی در سال تحصیلی 96-1395بود. تعداد نمونه بر اساس جدول نمونه ­گیری مورگان ۲۸۸ نفر بوده که به روش تصادفی خوشه­ ای (چند مرحله­ ای) انتخاب شدند. ابزارهای پژوهش پرسشنامه افسردگی بک و اعتیاد به اینترنت یانگ بوده است. برای تجزیه و تحلیل داده ها از نرم افزار spss-21و برای تحلیل داده­ ها از آزمون­ های آماری t، تحلیل واریانس یک طرفه و ضریب همبستگی اسپیرمن استفاده گردید. یافته ­ها: نتایج پژوهش نشان داد که بین اعتیاد به اینترنت و افسردگی دانش­آموزان، رابطه مثبت و معناداری وجود (r=0.324; P<0.001)، همچنین بین دختران و پسران رابطه معناداری مشاهده نشد. بیشترین میزان استفاده از شبکه ­های مجازی نیز از تلگرام (با میانگین 40.29)، سپس هر دو (با میانگین 39.53) و در نهایت از اینستاگرام (با میانگین 37.80) بوده است. نتیجه­ گیری: با توجه به وجود ارتباط معنادار بین اعتیاد به اینترنت و افسردگی بین دانش ­آموزان، توصیه می ­شود برنامه های آموزشی برای دانش ­آموزان، اولیای مدرسه و والدین طراحی و اجرا گردد.

Title: سواد سلامت کارمندان دانشگاه علوم پزشکی همدان و عوامل دموگرافیک مرتبط با آن

http://jech.umsha.ac.ir/article-1-389-fa.html

سابقه و هدف: یکی از تعیین کننده های مهم سلامت در هر جامعه ای، سواد سلامت است. مطالعه حاضر با هدف بررسی وضعیت سواد سلامت کارمندان دانشگاه علوم پزشکی همدان و عوامل دموگرافیک مرتبط با آن صورت گرفت. مواد و روش ها: پژوهش حاضر یک مطالعه مقطعی از نوع توصیفی- تحلیلی بود که در سال 1395 در 188 نفر از کارمندان دانشگاه علوم پزشکی همدان انجام شد. کارمندان از طریق روش نمونه گیری طبقه ای وارد مطالعه شدند. ابزار گردآوری اطلاعات، پرسشنامه سواد سلامت بزرگسالان ایرانی (HELIA) بود که از طریق خودگزارش دهی تکمیل گردید. داده ها با استفاده از آزمون های کای دو، ضریب همبستگی، آنالیز واریانس یکطرفه و رگرسیون خطی با استفاده از  نرم ­افزار SPSS  نسخه 22 مورد تجزیه و تحلیل قرار گرفت. یافته ­ها: میانگین سنی کارمندان 36.84 سال بود. اکثر کارمندان (74.5 درصد)، اطلاعات مرتبط با سلامت را از طریق اینترنت کسب می کردند. در بین ابعاد پنجگانه سلامت، بعد فهم اطلاعات سلامت از وضعیت مطلوبتری برخوردار بود. بالاترین ضریب همبستگی بین ابعاد سواد سلامت مربوط به ارتباط بین فهم و تصمیم گیری بود (0.05>, P0.582=r). به طور متوسط سواد سلامت کارمندان در سطح مرزی بود و سن، وضعیت تأهل، وضعیت تحصیلات و منابع کسب اطلاعات پیشگویی کننده سواد سلامت در کارمندان بودند. نتیجه ­گیری: در مجموع نتایج مطالعه نشان داد سواد سلامت کارمندان در حد مرزی بود.  به نظر می رسد جهت بهبود وضعیت سواد سلامت کارمندان، لازم است مداخلاتی مبتنی بر ویژگی های دموگرافیک طراحی و اجرا گردد.

Title: ارتباط مال اکلوژن با کیفیت زندگی مرتبط با سلامت دهان و دندان در نوجوانان پسر دبیرستانی شهرستان اهواز

http://jech.umsha.ac.ir/article-1-291-fa.html

سابقه و هدف: امروزه یکی از موارد قابل توجه در حیطه­ی دندانپزشکی، تأثیر شرایط دهان و دندان بر کیفیت زندگی و فعالیتهای روزانه بیماران می باشد. این مطالعه با هدف بررسی ارتباط مال اکلوژن با کیفیت زندگی مرتبط با سلامت دهان و دندان در نوجوانان پسر دبیرستانی شهر اهواز، انجام گرفت. مواد و روش ها: در این مطالعه ای توصیفی-تحلیلی و مقطعی، 192 دانش آموزان پسر دوره متوسطه با سن 18-14 سال به روش خوشه ای چند مرحله ای تصادفی در سال 1395 انتخاب و در مطالعه شرکت ابزار گردآوری اطلاعات شامل؛ پرسشنامه اطلاعات دموگرافیک و پرسشنامه های استاندارد تاثیرات سلامت دهانی Oral Impact on Daily Performance (OIDP)) به همراه  شاخص نیاز به درمان ارتودنسی  Index of Orthodontic Treatment Need (IOTN) بود. داد ها با نرم افزار SPSS 16 و آزمون های توصیفی و تحلیلی آنالیز شدند. یافته ها: میانگین کیفیت زندگی کلی نمونه های این مطالعه، 6.48 با انحراف معیار 19.47  بود و در مجموع، وضعیت دهان و دندان 26.6 درصد نمونه ها  بر کیفیت زندگی آنان تاثیر داشت. بیشترین فعالیت متأثر شده از وضعیت دهان و دندان با 15.6 درصد، مربوط به لبخند زدن بوده و پس از آن شرایط احساسی با 9.9 درصد قرار داشت. ارتباط بین نمره کیفیت زندگی مرتبط با سلامت دهان با  جزء سلامت دندانی شاخص IOTN معنی دار نبود (p= 0.13)، اما ارتباط معنی داری بین نمره کیفیت زندگی مرتبط با سلامت دهان بر اساس جزء زیبایی شناختی، وجود داشت (p< 0.004). نتیجه­ گیری: با توجه به اهمیت سلامت دهان و دندان در ارتقاء کلی کیفیت زندگی افراد و از آنجاییکه نتایج این مطالعه نشان داد که این بیماران در وضعیت مناسبی از نظر کیفیت زندگی قرار ندارند، مدنظر داشتن جنبه های مختلف اجتماعی و روانی وضعیت دهان و دندان در ارائه­ی خدمات دندانپزشکی، به منظور ارتقاء کلی کیفیت زندگی افراد، توصیه می گردد.

Title: ارتباط سواد سلامت و میزان اعتیاد در زنان سنین باروری مراجعه کننده به مراکز ترک اعتیاد شهر تهران

http://jech.umsha.ac.ir/article-1-364-fa.html

سابقه و هدف: با توجه به تاثیر سواد سلامت بر ابعاد جسمی، روحی و کیفیت زندگی افراد، هدف از انجام پژوهش حاضر تعیین ارتباط سواد سلامت و میزان اعتیاد در زنان سنین باروری شهر مراجعه کننده به مراکز ترک اعتیاد شهر تهران بود. مواد و روش ها: پژوهش حاضر یک مطالعه مقطعی از نوع توصیفی - تحلیلی بود. جامعه آماری مطالعه، شامل تمامی زنان سنین باروری بود که در شش ماه اول سال 1396 به مراکز ترک اعتیاد شهر تهران مراجعه کرده بودند. از این میان 141 نفر به روش سرشماری و در دسترس انتخاب و وارد مطالعه شدند. ابزار مطالعه شامل پرسشنامه دو بخشی اطلاعات دموگرافیک و سواد سلامت بزرگسالان ایرانی بود. برای سنجش اعتیاد زنان نیز فراوانی مواد مخدر مصرفی افراد مدنظر قرار گرفت. در این پژوهش از نرم افزار آماری SPSS نسخه 20  و همچنین از آزمون های آماری پارامتریک و آزمون ANOVA برای تجزیه و تحلیل داده های مطالعه استفاده شد.  یافته ها: میانگین سواد سلامت کل شرکت کنندگان در مطالعه 65.11 به دست آمد. از بین ابعاد سواد سلامت، بعد ارزیابی با 31.5 درصد و بعد درک و فهم با 17.82 درصد به ترتیب بیشترین و کمترین درصد میانگین از حداکثر نمره قابل اکتساب را از بین ابعاد مختلف سواد سلامت کسب کردند. نتایج حاصل از این مطالعه نشان داد میان سواد سلامت و میزان اعتیاد در زنان سنین باروری ارتباط مثبت معنی داری وجود دارد. نتیجه گیری: به نظر می رسد سواد سلامت یکی از ارکان اصلی سلامت در میان زنان محسوب می شود و می تواند تبعات مثبتی بر زندگی زنان داشته باشد، تا آنجا که در این مطالعه مشخص گردید زنان با سواد سلامت بالاتر گرایش کمتری به اعتیاد دارند.

Title: سواد سلامت در زنان مراجعه کننده به مراکز بهداشتی درمانی شهر زنجان: یک مطالعه مقطعی

http://jech.umsha.ac.ir/article-1-354-fa.html

سابقه و هدف: سواد سلامت شامل مجموعه ای از مهارت هاست که فرد را قادر به خواندن، شنیدن و تجزیه و تحلیل و تصمیم گیری و بکارگیری این مهارت ها در مسائل مربوط به سلامت می نماید که وجود این مهارت برای زنان در یک خانواده در سلامت و شادابی اعضای خانواده و کل جامعه تاثیر به سزائی دارد. هدف از مطالعه حاضر تعیین سطح سواد سلامت زنان مراجعه کننده به مراکز بهداشتی درمانی شهری تحت پوشش مرکز بهداشت شهرستان زنجان در سال 1395 بود. مواد و روش ها: پژوهش حاضر یک مطالعه توصیفی-تحلیلی و از نوع مقطعی بود که در بین 323 نفر از زنان مراجعه کننده به مراکز بهداشتی درمانی شهر زنجان که به روش نمونه گیری تصادفی طبقه ای انتخاب شده بودند، انجام گردید. ابزار جمع آوری داده ها پرسشنامه سواد سلامت عملکردی بالغین بود. داده های این مطالعه با استفاده از نرم افزار SPSS نسخه 21 مورد تجزیه و تحلیل قرار گرفت. یافته ها: میانگین سنی شرکت کنندگان 5.4±30.8 سال بود و اکثر افراد (39 درصد) تحصیلات دانشگاهی داشتند. 22.6 درصد از افراد مورد مطالعه سواد سلامت ناکافی، 22.3 درصد سواد سلامت مرزی و 50.1 درصد سواد سلامت کافی داشتند. سواد سلامت ارتباط معنی داری با سطح تحصیلات (P<0.001) و وضعیت شغلی شرکت کنندگان در مطالعه  داشت (P=0.044). نتیجه گیری: نتایج این مطالعه نشان داد سواد سلامت حدود نیمی از زنان مراجعه کننده به مراکز بهداشتی درمانی شهر زنجان در حد مطلوب نبود.  نظر به نقش حیاتی بانوان در سلامت خانواده و اجتماع ضروری است تا برنامه ریزی آموزشی در زمینه ارتقاء سواد سلامت این گروه مهم انجام شود.

Title: اثرات ازدحام ترافیک شهری سنندج بر تعیین کننده های اجتماعی سلامت از دیدگاه ساکنین و مطلعین کلیدی: یک مطالعه کیفی

http://jech.umsha.ac.ir/article-1-279-fa.html

سابقه و هدف: حمل و نقل و ترافیک که امروزه به عنوان یک پدیده سیاسی- اجتماعی نقش مهمی در کیفیت و ساختار اقتصادی – اجتماعی جوامع ایفا می نماید، یکی از بنیان های زندگی نوین شهری و نیازهای اجتماعی انسان را شکل می دهد. هدف از مطالعه حاضر تبیین دیدگاه های ساکنین شهر و مطلعین کلیدی از اثرات ترافیک و حمل و نقل درون شهری سنندج بر تعیین کننده های اجتماعی سلامت بود. مواد و روش ها: مطالعه بصورت کیفی و به روش تحلیل محتوی قراردادی انجام شد. شرکت کنندگان در پژوهش 22 نفر از ساکنین شهر و 8 نفر مطلع کلیدی بودند که به ترتیب در جلسات مصاحبه گروهی متمرکز (4 جلسه) و مصاحبه عمیق فردی مورد مصاحبه قرار گرفتند. روش نمونه گیری مبتنی بر هدف بود. جهت مدیریت داده ها از نرم افزار MAXQDA10 استفاده شد. یافته ها: مؤلفه های اجتماعی سلامت که از دید شرکت کنندگان تحت تأثیر ازدحام ترافیک شهری در سنندج بودند شامل عوامل اجتماعی/فرهنگی (تفریح و اوقات فراغت، روند کاری، امنیت رفت و آمد، مصرف دخانیات و آموزش و پرورش) و عوامل محیطی (پوشش گیاهی، آثار باستانی، وضعیت خاک،  غذا و آب، آلودگی صوتی، وضعیت زیبایی شناختی شهری، و محیط زیست بطور کل) بود. نتیجه گیری: دست اندرکاران و سیاستگذاران سیستم ترافیک شهری و نیز متخصصین حوزه سلامت در ایران باید توجه بسیار بیشتری به اثرات طرحها و برنامه های ترافیکی بر سلامت و تعیین کننده اجتماعی آن داشته باشند. ارزیابی جامع اثرات ترافیک و حمل و نقل شهری بر سلامت و تعیین کننده های آن در کشور ضروری بنظر می رسد.

Title: تاثیر کلاس های آمادگی برای زایمان در توانمندی مادران باردار: یک مرور نظام مند

http://jech.umsha.ac.ir/article-1-450-fa.html

سابقه و هدف: بارداری یکی از مهم ترین مراحل زندگی زنان می باشد که منجر به تغییرات جسمی، روانی و اجتماعی قابل ملاحظه‌ای می‌گردد. لذا توانمند سازی مادران باردار جهت سازگاری با این تغییرات ضروری بنظر می‌رسد. از آنجائی که کلاسهای آمادگی برای زایمان به همین منظور طراحی گردیده اند، مطالعه حاضر با هدف مروری برمطالعات انجام شده در زمینه تاثیر کلاس های آمادگی برای زایمان بر توانمندی مادران باردار در ایران انجام گرفت. مواد و روش ها: با استفاده از کلمات کلیدی در 12 پایگاه های اطلاعاتی معتبر و موتور جستجوی Google Scholar، مقالات مرتبط منتشر شده تا ابتدای آوریل 2017 انتخاب و مورد بررسی قرار گرفت. در مجموع 96 مقاله یافت شد. چکیده و متن کامل مقالات مرتبط برای تعیین شرایط ورود به مطالعه بررسی شدند. در نهایت 10 پژوهش وارد مطالعه مروری شدند. یافته ها: نتایج حاکی از آن بودکه خستگی، درد کمر و لگن و فعالیت روزانه در دوران بارداری، افسردگی پس از زایمان و مدت بستری در بیمارستان بین دو گروه تفاوت آماری معناداری را نشان داده اند. همچنین در مدت فاز فعال زایمان و مرحله دوم زایمان، شدت اضطراب، خود کارآمدی، وزن نوزاد، میزان رضایت و میزان زایمان طبیعی در بین دو گروه، اختلاف آماری معناداری مشاهده شد. در مجموع 81.46 درصد گروه مداخله و 55.88 درصد گروه کنترل، زایمان طبیعی داشته اند. نتیجه گیری: بررسی مقالات نشان می دهد آموزش های کلاسهای آمادگی برای زایمان در توانمند سازی مادران باردار موثر بوده است. لذا تشویق مادران به شرکت فعال در این کلاسها و توسعه کلاسهای آمادگی برای زایمان در سطح کشور توصیه می شود.
